# Understanding and engineering receptor–ligand interactions

**DOI:** 10.1111/tpj.71135

**Published:** 2026-09-22

**Authors:** Verónica G. Doblas, Isabel Monte, Elwira Smakowska‐Luzan, Adam D. Steinbrenner

**Affiliations:** ^1^ Instituto de Hortofruticultura Subtropical y Mediterránea La Mayora (IHSM‐UMA‐CSIC) Málaga Spain; ^2^ ZMBP, University of Tübingen Tübingen Germany; ^3^ Laboratory of Biochemistry Wageningen University & Research Wageningen the Netherlands; ^4^ Department of Biology University of Washington Seattle Washington USA

## DISSECTING LIGANDS, RECEPTORS, AND SIGNAL TRANSDUCTION

Plants perceive cues from their internal and external environment and activate context‐appropriate responses. The sensors in these processes are receptors: proteins able to perceive specific ligands to transduce appropriate cellular signals. They are essential for mediating plant growth, development, and stress responses. The field of receptor–ligand interactions has grown enormously in recent years. We have moved from defining and characterizing key receptors for classical ligands, such as plant hormones in growth pathways and pathogen‐derived elicitors in defense pathways, to a far greater understanding of diverse ligand–receptor networks, complex spatiotemporal regulation, and downstream signaling functions, often in non‐traditional model organisms. In this special issue, a series of timely reviews highlights new and emerging themes in receptor–ligand interactions, as well as new depth regarding classical receptor pathways. By understanding molecular mechanisms of receptor function, the engineering of recognition and signaling networks becomes feasible.

## FROM PRECURSOR TO PERCEPTION: LIGAND PROCESSING AND MOLECULAR RECOGNITION

Cell‐surface receptors perceive peptides derived from plant‐interacting organisms, damaged plant cells, or plant cells actively releasing such peptides during development or in response to environmental perturbations. In many cases, protein precursors require the action of peptidases to release peptide ligands. Subtilases are peptidases that process peptide ligands involved in development or immunity. Matsui and Hirakawa reviewed how different peptide families are cleaved by subtilases and discussed both subtilases' specificity and regulation. In addition to subtilases, papain‐like cysteine proteases and metacaspases are also involved in the proteolytic cleavage and release of peptides described as phytocytokines (Matsui & Hirakawa, 2026). Conceptually analogous to animal cytokines, phytocytokines are small‐secreted peptides with immunity modulation functions. Koenig et al. compare how phytocytokines and cytokines are processed by distinct proteases, released, and secreted to the extracellular space, where they are perceived by specific receptors to modulate immune responses (Koenig et al., 2026). Due to their role in immunity, phytocytokines are targets for plant‐interacting microbes that sometimes exploit phytocytokine mimicry to manipulate the plant host signaling pathways.

Upon processing and release of self‐ and non‐self‐ligands, plants employ cell‐surface receptors for ligand perception and initiation of downstream signaling. Ngou et al. describe how ligand perception by the receptors induces the association with phylogenetically related components to activate distinct downstream signaling pathways (Ngou et al., 2026). Notably, receptor‐like kinases (RLKs) share a common evolutionary origin, and their diversification and modular evolution enabled receptors to acquire new functions. Pathogen pressure probably led to the diversification of receptors involved in immunity, whereas receptors regulating key developmental processes remained mostly conserved. The majority of receptor–ligand pairs characterized so far correspond to leucine‐rich repeat (LRR) and LysM receptors perceiving peptides or N‐acetylated glycans, respectively. Yet, glycan perception is also mediated by receptors with alternative architectures such as lectins, cysteine‐rich epidermal growth factor (EGF) motif (present in WALL‐ASSOCIATED KINASES, WAKs, and WAKs‐like), LRRs, LRR‐malectin and malectin‐like LRR. Vílchez‐Pinto et al. focus on how glycans are recognized by plant and animal immune receptors, highlighting conserved and divergent mechanisms in plant and animal immunity (Vílchez‐Pinto et al., 2026). Unlike peptide receptors, glycan receptors can detect different ligand features, including their topology or linkages rather than the individual glucose units. Furthermore, the diversity of glycan recognition by different receptor families entails diverse protein‐carbohydrate molecular interactions, underscoring the need for structural analyses of additional glycan‐receptor pairs.

## CLASSIC TOPICS, NEW FINDINGS: HORMONE AND SELF‐INCOMPATIBILITY SIGNALING

Hormone signaling has long been a classical topic in the field of receptor–ligand interaction. Among plant hormones, brassinosteroids (BRs) have been extensively studied, particularly through their perception by the LRR RLK receptor BRASSINOSTEROID‐INSENSITIVE 1 (BRI1) and its homologs. Decades of research have established BR signaling as one of the best‐characterized signaling pathways in plants. Yet, recent advances are revealing an increasingly complex picture in which hormone perception and signaling are highly dynamic, spatially restricted, and context‐dependent.

In this special issue, Blanco‐Touriñán et al. review how BR perception is predominantly cell‐autonomous and varies according to cellular and developmental context (Blanco‐Touriñán & Hardtke, 2025). Emerging technologies are providing new insights into the spatial organization of BR signaling. Single‐cell transcriptomic analyses in Arabidopsis roots, for example, have revealed that BRs regulate distinct transcriptional programs across different spatiotemporal contexts and that BR signaling is required in multiple tissues for normal root growth. The review also highlights how BR signaling is integrated with other receptor‐mediated pathways, including CLE and PSK peptide signaling, as well as auxin signaling, to coordinate processes such as vascular patterning.

Aiba et al. further illustrate how the regulation of BR signaling extends beyond ligand perception to the dynamic control of receptor abundance and organization at the plasma membrane (Aiba et al., 2026). BRI1 undergoes internalization through several partially overlapping endocytic routes, providing robust control of receptor levels at the cell surface. Environmental cues, including elevated ambient temperature, can also promote BRI1 endocytosis and degradation. In addition, BRI1 clustering and nanodomain formation at the plasma membrane emerge as important regulatory mechanisms. These processes are closely connected to cell‐wall status, which can influence the formation of membrane protein clusters. The RALF–FER–LLG1 signaling module, for instance, promotes endocytic hotspots that facilitate the internalization of multiple receptors, including BRI1 and FLS2, linking cell‐wall integrity to the spatial organization of receptor signaling.

Self‐incompatibility (SI) provides another example of a classical signaling system that is being redefined by recent discoveries. SI is a tightly regulated cell–cell communication mechanism that prevents self‐fertilization in flowering plants and has traditionally been exemplified by the Brassicaceae system, in which the RLK SRK perceives its cognate cysteine‐rich peptide ligand, SCR/SP11. In this special issue, Lin et al. review how SI systems in the Papaveraceae, Poaceae, and Convolvulaceae rely instead on membrane proteins lacking the canonical features of RLKs (Lin et al., 2026). Despite their structural diversity, these systems frequently use highly polymorphic cysteine‐rich proteins as ligands, pointing to convergent evolutionary solutions for self/non‐self‐recognition. By integrating genetic, biochemical, structural, and evolutionary evidence, the review introduces the concept of atypical receptors (ATRs) as central components of plant reproductive signaling. These findings expand the classical view of receptor‐mediated signaling and highlight the remarkable diversity of molecular strategies that plants have evolved to perceive and discriminate between self and non‐self.

## ENDOGENOUS LIGAND–RECEPTOR MODULES IN DEVELOPMENT, STRESS, AND CELL‐WALL SURVEILLANCE

Plants must constantly balance growth and defense mechanisms to withstand environmental challenges. The work of Chen et al., Zecua‐Ramirez et al., and Zwartkruis et al. demonstrates that endogenous peptide–receptor modules are central to this balance. Rather than acting as isolated developmental or stress signals, peptide pathways provide flexible molecular linkages that connect cellular status, tissue integrity, and environmental perception with decisions about growth.

The research article by Chen et al. describes a mechanistic pathway by which the phytocytokine peptide SCOOP12 restricts Arabidopsis root meristem activity (Chen et al., 2025). Perception of SERINE‐RICH ENDOGENOUS PEPTIDE 12 (SCOOP12) by the RLK MALE DISCOVERER 1‐INTERACTING RECEPTOR‐LIKE KINASE 2 (MIK2) requires MPK6 and decreases the abundance of the stem‐cell regulators PLETHORA1 (PLT1) and PLT2. MPK6 interacts with and phosphorylates PLT1 and PLT2, while SCOOP12‐induced hydrogen peroxide accumulation promotes their proteasome‐dependent degradation. Reduced PLT activity correlates with decreased mitotic activity and a lower number of meristematic cells. This provides a mechanistic explanation for how immune‐related signaling inhibits root growth. Findings presented in this article extend the established SCOOP–MIK2 pathway, previously implicated in immunity, cell‐wall integrity, and auxin transport, to include direct regulation of root stem‐cell‐niche homeostasis (Hou et al., 2021; Rhodes et al., 2021; Wang et al., 2024).

The focused review by Zecua‐Ramirez et al. places the SCOOP–MIK2 signaling mechanism within the broader landscape of peptide‐mediated signal integration. CLAVATA 3/EMBRYO SURROUNDING REGION (CLE), C‐TERMINALLY ENCODED PEPTIDE (CEP), PHYTOSULFOKINE (PSK), ROOT MERISTEM GROWTH FACTOR (RGF), PLANT ELICITOR PEPTIDE (PEP), RAPID ALKALINIZATION FACTOR (RALF), and SCOOP peptides regulate developmental patterning, nutrient acquisition, abiotic stress adaptation, and interactions with pathogenic or beneficial microbes (Zecua‐Ramirez et al., 2026). Bai and Torii reveal similar themes for the diverse EPIDERMAL PATTERNING FACTOR (EPF/EPFL) peptide family, perceived by ERECTA receptors at the cell surface (Bai & Torii, 2026). Beyond classical stomatal development phenotypes, network specificity in recognition as well as spatial patterning and different signaling functions of ER receptors can generate diverse developmental outputs. Notably, diverse ligand–receptor pairs often converge on shared components, including SOMATIC EMBRYOGENESIS RECEPTOR KINASE (SERK) co‐receptors, receptor‐like cytoplasmic kinases, calcium signaling, reactive oxygen species, and MAP kinase cascades. An unresolved question, therefore, is how signaling specificity is ultimately achieved. Ligand abundance, receptor composition, cellular location, and timing are likely crucial. The antagonistic actions of SCOOP10 and SCOOP12 via MIK2 offer an instructive example of how peptide mixtures can precisely regulate a shared receptor pathway and its developmental consequences (Zhang et al., 2024).

Finally, Zwartkruis et al. focus on THESEUS1 (THE1), a CrRLK1L receptor kinase that connects cell‐wall perturbation with adaptive growth, hormonal, and defense responses (Zwartkruis et al., 2026). THE1 contributes to responses to impaired cellulose biosynthesis, including reactive oxygen species production, jasmonate accumulation, ectopic lignification, and growth inhibition. Moreover, THE1‐dependent induction of PROSCOOP18 provides a molecular connection between cell‐wall surveillance and MIK2‐mediated phytocytokine signaling (Zhai et al., 2024). Therefore, changes in cell‐wall integrity at a local level can be converted into peptide signals that regulate responses in areas beyond the originally affected cells.

## NEW DIRECTIONS IN PLANT RECEPTOR BIOLOGY

Volatile organic compounds (VOCs) include various small molecule volatiles, terpenes and terpenoids, ethylene, and methyl esters of jasmonic acid and salicylic acid. They are especially suited to potential plant–plant communication, but there are very few receptors known to perceive specific VOC compounds. In this issue, Bergman et al. review current knowledge of VOC receptors for MeSA, MeJA, ethylene, and karrikins (Bergman et al., 2026). They especially highlight the recent exciting discovery of an α/β‐hydrolase, the KARRIKIN INSENSITIVE 2 homolog PhKAI2ia from petunia as a receptor for the common sesquiterpene, (−)‐germacrene D. An overview of ligand types for the large KAI2 gene family suggests future directions for exploring novel derivatives, such as sesquiterpene lactones, as potential ligands.

With an abundance of available genomes, placing canonical receptor pathways in a broader phylogenetic and functional context is a growing theme. Fougner‐Økland et al. provide an extensive overview of new findings for LysM receptors in recognition of diverse fungal and bacterial oligosaccharides (Fougner‐Økland et al., 2026). A range of short‐chain and long‐chain oligosaccharides interact with members of the diverse LysM‐RK family to mediate either symbiosis or immunity responses. An increasing number of LYK and LYR subfamilies of LysM‐RKs have now been characterized in legume and non‐legume species, providing insight into the evolution of more specialized responses. Networks of receptors perceive different polysaccharides, and a given interaction will involve a mixture of oligosaccharides and host‐specific binding affinities and receptor complex dynamics.

A goal of understanding receptor–ligand interactions is the ability to deploy this knowledge in crop plants. Torres Ascurra et al. explore this theme for plant immune receptors, where modularity and specificity have generated great excitement in their deployment for disease resistance (Torres Ascurra et al., 2026). To combat potato disease, the authors highlight a wealth of known cell‐surface and NLR receptors from wild relatives in the *Solanum* section Petota, the more distantly related tomato (*Solanum lycopersicum*), and receptors from other genera such as EFR from Arabidopsis. Understanding if these pathways are robust in potato against pathogens, especially in the face of potential pathway suppression by pathogen effectors, will be critical for receptor deployment.

As the special issue shows, there is great potential for engineering suggested by specificity at all steps in receptor signaling—from processing of ligand precursors, presence/absence of specific receptor homologs, and cell type specificity. Our ability to modify plants to engineer traits of interest will be enhanced by understanding many facets of receptor biology for immune, developmental pathways. Just as plants must monitor their environment, plant researchers must also closely monitor how new tools and technologies are regulated. The precise way in which the genes are engineered is under changing regulation by national governmental agencies. By understanding and deploying knowledge of receptor–ligand interactions, engineering crop plant traits will be increasingly feasible.
